# Minimizing unnecessary tax audits using multi-objective hyperparameter tuning of XGBoost with focal loss

**DOI:** 10.3389/frai.2025.1669191

**Published:** 2025-10-16

**Authors:** Ivan P. Malashin, Igor S. Masich, Vadim S. Tynchenko, Andrei P. Gantimurov, Vladimir A. Nelyub, Aleksei S. Borodulin

**Affiliations:** Artificial Intelligence Technology Scientific and Education Center, Bauman Moscow State Technical University, Moscow, Russia

**Keywords:** company data, tax compliance, anomaly detection, machine learning, XGBoost

## Abstract

This study presents a machine learning (ML) approach for detecting non-compliance in companies' tax data. The dataset, consisting of over one million records, focuses on three key targets: invalid addresses, invalid director information, and invalid founder information. The analysis prioritizes young companies (≤ 3 years old) with fewer than 100 employees, thereby improving class distributions and model effectiveness. A combination of binary classification techniques was employed, including benchmarked supervised learning models (XGBoost, Random Forest), anomaly detection methods (LOF, Isolation Forest), and semi-supervised learning using deep neural networks (DNNs) with unlabeled data. Given its computational efficiency, XGBoost was selected as the primary model. However, class imbalance persisted even among young companies, necessitating the integration of focal loss to improve classification performance. To further enhance accuracy while maintaining model interpretability, NSGA-II (Non-dominated Sorting Genetic Algorithm II) was used for multi-objective hyperparameter optimization of XGBoost. The objectives were to maximize ROC-AUC for improved predictive performance and minimize the number of trees to enhance interpretability. The optimized model achieved a ROC-AUC of 0.9417, compared to 0.9161 without optimization, demonstrating the effectiveness of this approach. Additionally, SHAP analysis provided insights into key factors influencing non-compliance, supporting explainability and aiding regulatory decision-making. This methodology contributes to fair and efficient oversight by reducing unnecessary inspections, minimizing disruptions to compliant businesses, and improving the overall effectiveness of tax compliance monitoring.

## 1 Introduction

The accuracy and reliability of company records are integral to effective regulatory compliance, especially in the areas of tax and business oversight (Bello et al., 2024; Rahman et al., 2024). With an increasing volume of data generated by LEs, identifying inaccuracies in key records, such as addresses, directors, and founders, presents challenges for tax authorities. Inaccurate or outdated information can lead to issues like misdirected tax inspections or facilitate fraudulent activities. Automating the process of detecting invalid data can help reduce administrative tasks and improve compliance processes.

The task of classifying company records is complicated by factors such as imbalanced class distributions (Huang et al., 2016), high-dimensional data (Johnstone and Titterington, 2009), and difficulties in establishing clear decision boundaries (Dobslaw and Feldt, 2024) between valid and invalid entries. Traditional classification (Wang, 2008) methods often struggle with these challenges, necessitating the exploration of more advanced approaches capable of handling the complexities of real-world data.

This paper proposes a pipeline for the classification and validation of companies' records through the integration of supervised learning, anomaly detection, and semi-supervised learning techniques. The study applies deep semi-supervised learning (DSSL) (Oliver et al., 2018) combined with the feature-injected anomaly detection (FIAD) (Chen et al., 2025) framework to refine feature engineering for the classification of LE. Existing approaches to anomaly detection in tax compliance often struggle with incomplete or weakly labeled data and provide limited interpretability (Rahman et al., 2024; Liang et al., 2025; Ramesh et al., 2025; Mahesar et al., 2025), which reduces their applicability in real-world audit contexts. To address this gap, the present methodology aims to support the identification of potentially non-compliant companies that may engage in activities such as fraud, money laundering, or tax evasion. The overarching research question guiding this work is: *How can a tailored machine learning pipeline effectively detect anomalies in company records to reduce unnecessary tax audits while maintaining interpretability and generalizability?*

The study utilizes a dataset from the Federal Tax Service of Russia, which includes approximately one million records for the first three quarters of 2024. The dataset contains attributes related to LEs, including company addresses, director and founder details, tax registration information, and operational characteristics. The proposed pipeline's performance is evaluated through a series of experiments designed to address issues like class imbalance and data complexity.

The following sections detail previous research (Section 2, the methodology used to process and analyze the dataset (Section 3.1), the machine learning (ML) models tested (Section 3.2), and the results (Section 4) obtained from applying these techniques to the validation of company records. Additionally, the implications of the findings are discussed (Section 5), with a focus on improving tax authority inspections and identifying areas for further research.

## 2 Related work

Financial institutions face strict international regulations requiring rigorous efforts to prevent services from being exploited by criminals and terrorists. Current Anti-Money Laundering (AML) systems rely on watch-list filtering but often produce many false positives, requiring significant human intervention. (Alkhalili et al. 2021) introduces ML-based component to enhance watch-list filtering systems. It uses historical transaction data to analyze blocked transactions and provide recommendations, following a phased approach: monitoring, advising, and gradual action. It reduces manual workload by prioritizing high-risk cases while maintaining accuracy. Results show the ML-Component can streamline operations, cut compliance costs, and strengthen defenses against financial fraud.

Financial institutions are obligated to adhere to international regulations to prevent providing services to criminals or terrorists, with continuous monitoring of financial transactions necessary to detect suspicious activities. Businesses must ensure they validate customer information against reliable sources that confirm their identities or flag inconsistencies. Failure to detect fraudulent or suspicious transactions can result in harmful consequences, including fines or warnings for the financial institution. AML software, sanctions screening, and watch-list filtering are used to monitor transactions and ensure they do not involve prohibited individuals. While ML has been explored for improving Know Your Customer (KYC) systems (Ostern and Riedel, 2021), its application to watch-list filtering systems has been limited due to compliance concerns. (Savić et al. 2022) proposes a model for automating the check of blocked transactions in watch-list filtering systems using ML techniques. The model aims to address the challenge of false positives, reduce the workload of compliance officers, and speed up transaction processing. Through experiments with ML algorithms, it was found that support vector machines (SVM) provided the most accurate predictions for transaction decisions.

Tax evasion refers to actions, whether legal or illegal, that lead to the non-payment or underpayment of taxes. Proper tax payment is necessary for maintaining public services, including healthcare, education, and infrastructure. This is important in developing countries like Brazil, where large agencies like SEFAZ-CE manage databases of over 300,000 active taxpayer companies. However, manual tax inspections are time-consuming and prone to human error due to the complexity of fraud indicators. To improve this process, (Matos et al. 2020) propose Alicia, a feature selection method that uses association rules, propositional logic, and graph centrality to identify the most relevant features for detecting tax fraud. Alicia operates in three phases: generating association rules, building a graph from these rules, and ranking features based on their importance using a novel measure called Fraud Feature Topological Importance (F2TI) (Huang et al., 2014). Extensive tests show that Alicia outperforms other feature selection methods with F-measure scores up to 76.88%.

Tax evasion through related party transactions (RPTTE) (Zhou et al., 2024a), is a significant issue that can undermine tax systems and create unfair competition. To address this, a system called TaxThemis was developed by (Lin et al. 2020) to help tax officers identify suspicious RPTTE groups. This system integrates data mining and visual analytics to analyze and detect such groups through tax-related data. It builds networks of taxpayers and trade relationships, visualizing them to aid in the detection of RPTTE activities. The system includes modules for data preprocessing, analysis, and visualization, ensuring privacy while processing sensitive taxpayer information. TaxThemis enables interactive exploration, allowing officers to detect, analyze, and investigate suspicious groups through detailed visualizations and data features. Additionally, it incorporates advanced visual elements like calendar heatmaps and network diagrams to display tax-related transactions and profits, facilitating efficient investigations.

(Mousavi et al. 2022) evaluates the influence of corporate governance mechanisms, such as the characteristics of board members and audit committees, on fraud and money laundering in financial statements of firms listed on the Tehran Stock Exchange (Rostami et al. 2016) from 2014 to 2020. It uses descriptive correlation methodology with data from 154 firms across 27 industries, totaling 1,071 observations. Linear regression with panel data is applied, employing the Benish model for fraud measurement and auditors' opinions for money laundering. The results reveal that board and audit committee characteristics, including independence, financial and industry expertise, and effort, significantly reduce fraud and money laundering. The study suggests that corporate governance plays impact in improving financial statement integrity, providing useful insights for investors and policymakers. Additionally, tests such as normality, collinearity, and integration confirm the robustness of the data and models used.

With the growth of technology in Rwanda, the tax base has expanded, but this has also led to an increase in tax fraud. (Murorunkwere et al. 2023) focus on applying a supervised machine-learning models, including Artificial Neural Networks, Logistic Regression, and Random Forest, among others, to identify tax fraud. Findings show that businesses with certain characteristics, such as being registered for VAT, being involved in imports/exports, or located in the eastern province, are more prone to fraud. The data used in the study is anonymized, with 15,732 audited taxpayers from 2014 to 2019. Of these, 32.4% were found to have committed tax fraud, while 67.6% were non-fraudulent. The study utilized preprocessing techniques to handle imbalances in the data, including SMOTE and random under-sampling. The dataset was split into training, testing, and validation sets for optimal results.

(Xu et al. 2023) address corporate fraud prediction using ML, specifically leveraging the GONE framework. The research identifies fraud-related variables in four categories–Greed, Opportunity, Need, and Exposure—and applies ML models to predict corporate fraud in China. Among six models tested, the Random Forest (RF) model outperforms the others, with Exposure variables being the most significant predictors. The dataset is sourced from the China Stock Market and Accounting Research (CSMAR) (Zhang, 2024) and Chinese Research Data Services (CNRDS) (Feng and Nie, 2024) databases, including social media and news data. A sample of 35,922 firm-years from 2009 to 2018 reveals that about 12% of observations are related to fraud, similar to other studies on corporate fraud in China. The study categorizes fraud into more-serious and less-serious types and evaluates ML models based on metrics like AUC, precision, recall, and f1 score. The RF model shows the best performance, particularly in avoiding false-negative errors, which is a key factor for identifying fraudulent firms effectively.

In the field of applying ML to combat financial crimes and tax evasion, there is a range of studies that use models and approaches to enhance monitoring, filtering, and fraud prediction systems. These studies cover a wide array of methods, from improving AML systems to automating checks and detecting tax violations. Table 1 provides an overview of key research in this area, highlighting their focus, data used, applied models, and main results.

**Table 1:** Summary of related work.

**Reference**	**Focus**	**Data description**	**Applied model**	**Results**
Alkhalili et al. (2021)	Enhancing AML systems with ML	Historical transaction data, blocked transactions	ML-based component (monitoring, advising, gradual action)	Reduced manual workload, improved accuracy, cut compliance costs, strengthened fraud defenses
Savić et al. (2022)	Automating watch-list filtering	Blocked transaction data	Support vector machines (SVM)	SVM showed the most accurate predictions, reducing false positives and workload
Matos et al. (2020)	Tax fraud detection in Brazil	Database of 300,000+ active taxpayer companies in Brazil	Alicia (feature selection with association rules and graph centrality)	Alicia outperforms other feature selection methods with F-measure scores up to 76.88%
Lin et al. (2020)	Detecting tax evasion through RPTTE	Tax-related data, taxpayer and trade relationships	Data mining and visual analytics	identified suspicious RPTTE groups via network and visualization tools
Mousavi et al. (2022)	Corporate governance and fraud	Data from 154 firms across 27 industries (1,071 observations)	Linear regression with panel data	Corporate governance reduces fraud and money laundering, significant impact on financial integrity
Murorunkwere et al. (2023)	Predicting tax fraud in Rwanda	15,732 anonymized audited taxpayers (2014–2019)	Artificial neural networks, logistic regression, random forest	Identified fraud-prone characteristics, 32.4% of taxpayers committed fraud
Xu et al. (2023)	Corporate fraud prediction in China	Data from 35,922 firm-years (2009–2018) and social media/news data	Random Forest (RF), GONE framework	RF model outperformed others, with Exposure variables as most significant predictors

## 3 Materials and methods

### 3.1 Dataset description

**Figure 1:**
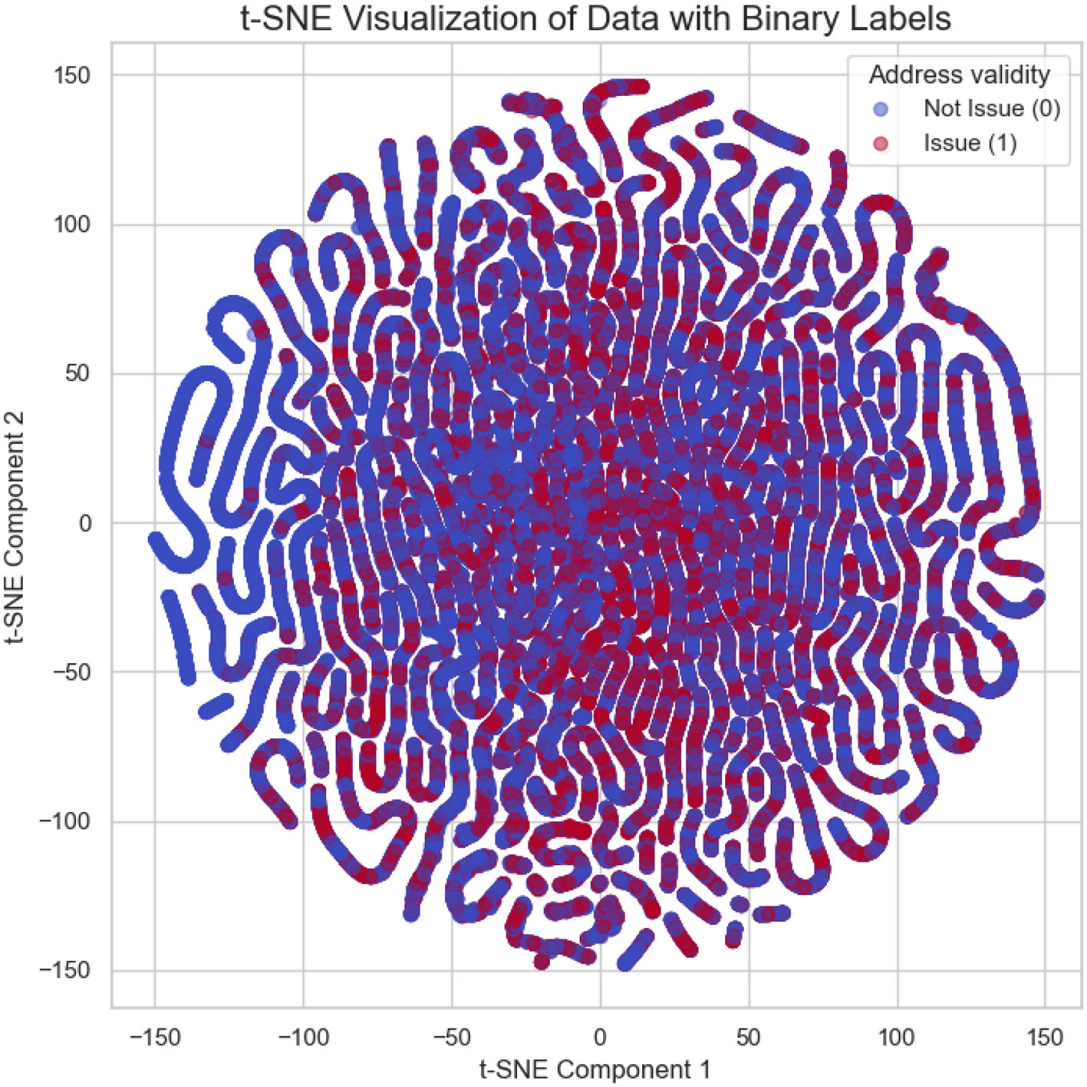
t-SNE visualization of companies based on feature space. Points blue denotes valid addresses, and red indicates entities with address-related issues.

One of the ideas was to assess the necessity of tax authorities conducting audits of companies based on their age. To this end, an analysis was performed on data collected for companies during the first three quarters of 2024. The dataset, provided by the Federal Tax Service of Russian Federation, included 1,008,725 tax audit records related to the accuracy of company addresses. These records were derived from data available to tax authorities, including information about real estate ownership by the LE's director or manager, the timeliness of submitted financial reports, and other relevant indicators. Of these, 678,795 records had no inaccuracies, while 329,930 were flagged as having invalid address information. Additionally, data on 924,364 directors of these companies were examined, with 678,795 directors having no invalid data and 42,681 flagged for inaccuracies. Information on 73,453 founders was also analyzed, revealing 42,681 founders with accurate data and 30,772 with invalid information. This analysis aimed to determine whether younger companies exhibit patterns that necessitate increased scrutiny, based on discrepancies in address and leadership data.

t-SNE visualization (Van der Maaten and Hinton, 2008) of company data is shown in Figure 1. Each point represents a LE, with the two components (t-SNE1 and t-SNE2) corresponding to reduced dimensions of the feature space. Colors indicate the validity of the entity's address: blue represents entities with valid addresses, while red corresponds to entities with address-related issues. The clear clustering patterns suggest underlying structural differences between the two groups, which may reflect distinct operational or compliance characteristics. The data is imbalanced (Hajibabaee et al., 2021), with a larger number of entities having valid addresses compared to those with address-related issues. Moreover, the t-SNE plot reveals overlapping, indicating poor separability between the two classes. These characteristics suggest that classification of such data poses challenges due to class imbalance and a lack of clear decision boundaries.

**Figure 2:**
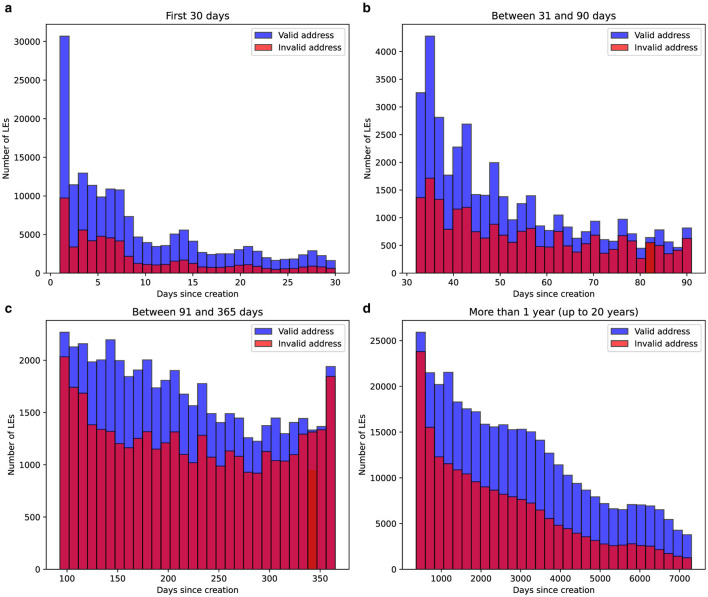
Distribution of the number of days since the creation of LEs, categorized by address validity. The histograms are divided into four time intervals: **(a)** first 30 days, **(b)** between 31 and 90 days, **(c)** between 91 and 365 days, and **(d)** more than one year (up to 20 years). Blue bars represent companies with valid addresses, while red bars indicate entities with invalid address records. The data highlights the differences in distribution between the two groups across age ranges of LEs.

The relationship between the age of companies and the likelihood of address validity issues was analyzed by examining the distribution of days since their creation. The dataset was segmented into four distinct time ranges: companies younger than 30 days, those aged 31 to 90 days, 91 to 365 days, and older than one year (up to 20 years). Histograms were plotted to visualize the proportion of entities with valid and invalid addresses in each interval. The results are summarized in Figure 2, highlighting the temporal dynamics of address validity concerns among LEs

The analysis shows clear patterns in the distribution of companies by age and address validity. During the first 30 days, many new companies are created, with a noticeable share flagged for invalid addresses. This trend continues at lower volumes in the 31-90 day range. By the time a company reaches one year and inspections are conducted, the number of entities with invalid addresses is much higher compared to other periods. After the first year, the number of invalid-address entities decreases significantly, likely due to attrition or corrections over time.

**Figure 3:**
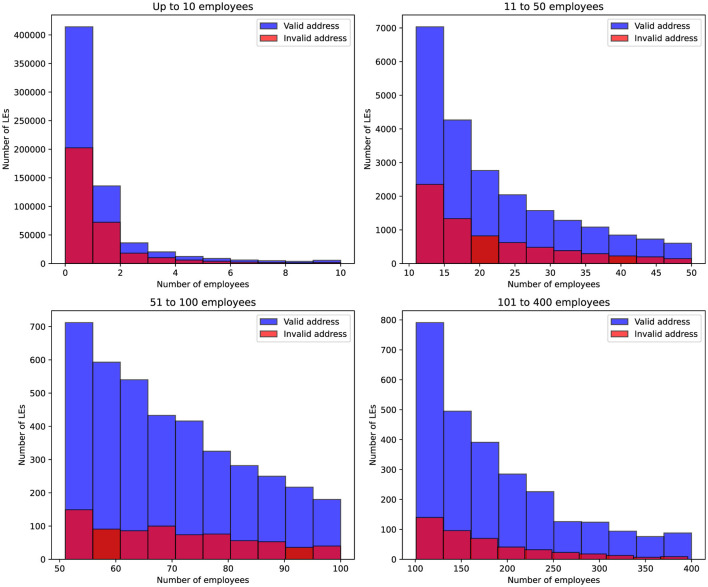
Distribution of companies based on their employee count and address validity. The blue bars represent companies with a valid address, while the red bars indicate companies with an invalid address. The data is grouped by four employee ranges: up to 10, 11–50, 51–100, and 101–400 employees.

Figure 3 presents the distribution of companies according to both their employee size and the validity of their registered address. The histograms show two categories: companies with a valid address and those with an invalid address, across four predefined employee ranges. The chart shows that companies with 1 to 2 employees most often have records with invalid addresses based on tax authority checks. However, for companies with more than 50 employees, there is a significant shift: among 700 companies in this category, fewer than 50 have invalid addresses

Since the dataset on inspections was too large, the goal was to remove records that did not significantly contribute to improving the identification of companies with invalid information. As part of this process, entities that have existed for more than 3 years and with more than 100 employers were excluded.

**Figure 4:**
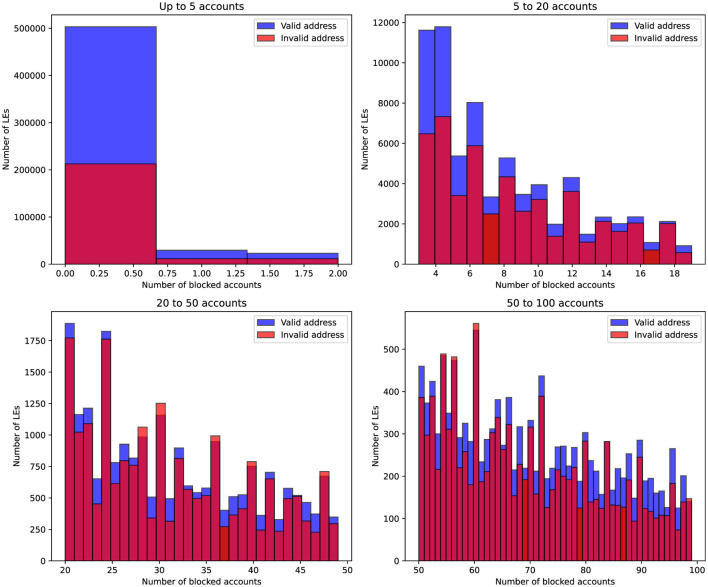
Distribution of companies based on their employee count and address validity. The blue bars represent companies with a valid address, while the red bars indicate companies with an invalid address. The data is grouped by four employee ranges: up to 10, 11–50, 51–100, and 101–400 employees.

Figure 4 visualizes the distribution of blocked accounts across different company sizes, segmented by the validity of the company's registered address. Companies are grouped into four account ranges, and the histograms illustrate how the frequency of blocked accounts varies between companies with valid and invalid addresses. It is evident that for companies with zero blocked accounts, less than 40% had incorrect address records according to the tax authority's verification results. However, as the number of blocked accounts increases, the proportion of companies with invalid address data significantly rises and begins to dominate

**Figure 5:**
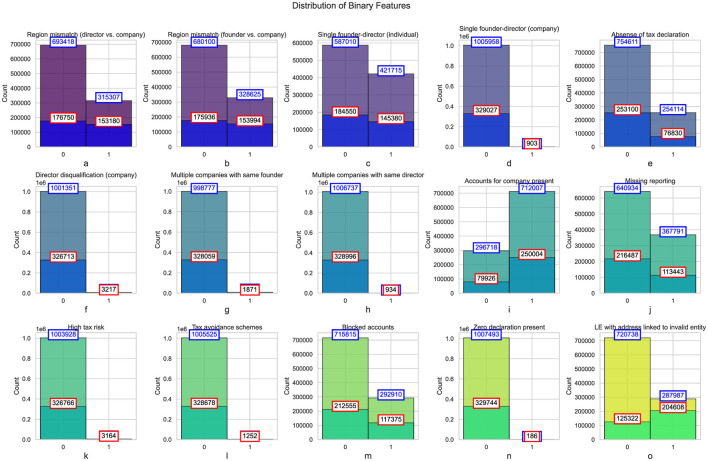
Histograms of binary features categorized by address validity indicator. Total number of companies in each category for each feature represented in blue, and the subset of companies marked with the address validity issue indicator shown in red.

Figure 5 presents histograms of binary features, categorized by the presence of the address validity indicator. The analysis reveals a discrepancy between the regions of directors and companies, as well as between founders and companies. In both cases, a substantial proportion of companies exhibit mismatches in regions, with approximately 31.5% of companies having a mismatch between the director and company regions, and 32.8% showing a mismatch between the founder and company regions. When focusing on companies with address validity issues, the proportion of mismatches increases notably. This discrepancy could indicate either an intentional attempt to obscure the entity's location or a failure to update company records accurately. Address mismatches are often associated with issues such as fraudulent reporting or non-compliance with local regulations, which may increase the risk of tax evasion or financial misconduct.

The data also shows a considerable number of companies with a single founder-director. While this is a common setup in smaller businesses, the prevalence of such structures warrants attention, particularly when examining discrepancies in the tax declarations of these entities. For instance, in the case of individual founder-directors, 42.2% of companies exhibited this structure, with a higher concentration among those subject to address validity issues (45.7%). A higher proportion of companies with singular founder-director structures may indicate a lack of corporate governance mechanisms, potentially making these entities more susceptible to non-compliance or financial mismanagement. Furthermore, these entities could be prone to issues like tax avoidance or underreporting of income, as the concentration of control in a single individual often leads to weaker oversight.

A significant proportion of companies (25.2%) were found to have no tax declarations filed (Figure 5e). This lack of compliance with tax regulations is of considerable concern, as it could suggest attempts to evade taxes or failures to comply with regulatory requirements. When the data is filtered to include only those entities with address validity issues, the proportion of companies without tax declarations increases to 23.3%. This could point to deliberate attempts to conceal financial activities or an indication of administrative negligence. The absence of tax declarations, especially in entities with address discrepancies, warrants closer scrutiny, as these are typically red flags for financial fraud or illegal activities.

Another finding is the number of directors disqualified (Figure 5f) from holding office in certain companies. Although the overall proportion of disqualified directors is low (less than 1%), the number increases among companies with address validity issues. This discrepancy could reflect the presence of entities that operate with directors who have a history of non-compliance, legal issues, or involvement in fraudulent activities. Director disqualification is an important measure to ensure corporate governance and mitigate risks related to financial misconduct. The higher incidence of disqualified directors in companies with address validity concerns suggests that these companies may be more prone to illicit activities or governance failures.

The data also highlights the prevalence of multiple companies being owned or directed by the same individual (Figure 5g). While only a small percentage of companies exhibit this pattern, it still raises concerns. The practice of founding multiple companies may indicate attempts to compartmentalize liabilities or evade tax audit. In entities subject to address validity issues, this practice is slightly more prevalent. The increased risk of fraudulent activity, such as tax avoidance, becomes more likely in these situations, as controlling multiple companies offers the opportunity for financial manipulation or the creation of artificial financial structures.

A concerning statistic is the high percentage of entities that are missing required reporting (Figure 5j) or deemed to present high tax risks (Figure 5k). Over 36.7% of all companies were found to have missing reporting, with a higher concentration among entities with address validity issues (34.3%). This missing reporting could be indicative of financial opacity, either intentional or due to inefficiencies in reporting practices. Similarly, a small but significant portion of entities (less than 1%) were categorized as presenting high tax risks. The higher incidence of these risks in companies with address validity issues could suggest a pattern of non-compliance or the involvement of companies in tax avoidance schemes.

The incidence of blocked accounts (Figure 5m) and zero tax declarations (Figure 5n). A substantial number of companies (29.3%) had blocked accounts, with a higher concentration among entities with address validity issues (35.5%). Blocked accounts are often associated with financial misconduct, such as money laundering or fraudulent activities, and their prevalence among companies with address discrepancies further indicates potential irregularities. Similarly, a small but concerning percentage of companies (0.1%) had zero tax declarations, which could indicate attempts to conceal financial activities or evade taxes.

Figure 5o highlights a clear link between address history and the likelihood of compliance issues among companies (Esayas and Mahler, 2015). For companies not linked to previously invalid entities, 28% (287,987 out of 1,008,725) have address issues, compared to 62% (204,608 out of 329,930) for those linked to invalid entities. This suggests that addresses associated with historically problematic entities carry elevated risks and are more likely to face regulatory scrutiny. Such patterns emphasize the need for stricter oversight of these addresses during company registration to mitigate fraud and ensure compliance. Businesses using such addresses may also encounter increased challenges due to reputational concerns.

The findings from this analysis suggest that a proportion of companies exhibit indicators of potential non-compliance or fraudulent activity (Kamoun et al., 2024). Entities with address validity issues tend to exhibit higher rates of discrepancies in region matching, tax reporting, and corporate governance. Possible reasons for these irregularities could include deliberate attempts to evade taxes or regulatory scrutiny, administrative negligence, or the lack of transparency in certain business structures.

The prevalence of mismatches in region, director disqualification, missing tax declarations, and other risk indicators points to the need for more stringent oversight and regulatory interventions. Address mismatches, in particular, should raise red flags for tax authorities and financial regulators, as they are often associated with attempts to obscure the true nature of a company's operations. Furthermore, the lack of tax reporting, high tax risk, and missing reporting signal areas where improvements in compliance and enforcement could reduce the overall risks associated with these LEs.

### 3.2 Pipeline

**Figure 6:**
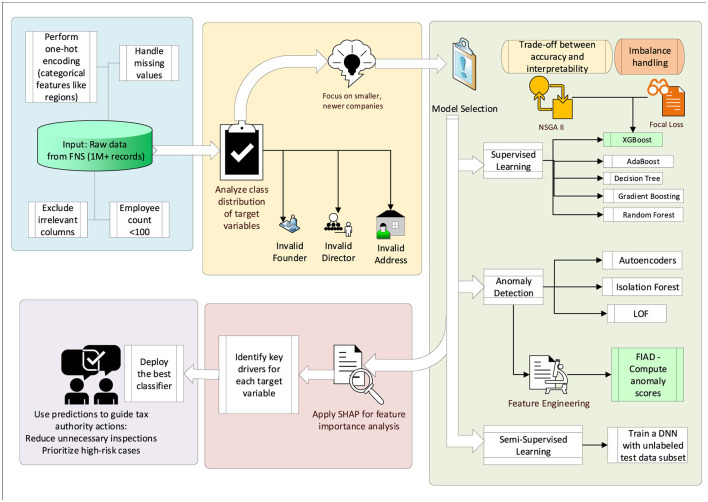
Experimental pipeline.

The study analyzed data on companies collected by the Federal Tax Service of Russia, encompassing approximately one million records for the first three quarters of 2024. The primary objective was to identify records with potential inaccuracies in three target variables: invalid companies addresses (current addresses linked to previously non-compliant LEs), invalid director information, and invalid founder information. The diagram of the proposed approach is shown in Figure 6.

Initial analysis revealed that the dataset was highly imbalanced and poorly separable. For instance, valid address records accounted for 678,795 entries, while invalid addresses totaled 329,930, constituting roughly one-third of the data. To address this, filtering was applied, focusing on younger companies (operating for about three years) with fewer than 100 employees. This refinement improved balance, yielding 433,415 valid and 168,770 invalid address records. Similarly, director-related records reduced from 843,214 valid and 81,150 invalid entries to 427,223 valid and 41,870 invalid, and founder-related records from 42,681 valid and 30,772 invalid to 21,065 valid and 19,732 invalid.

The problem was framed as a binary classification task with imbalanced classes (Cateni et al., 2014; Lee and Seo, 2022). Three methodological approaches were employed to address these challenges: supervised learning, anomaly detection, and semi-supervised learning.

In the supervised setting, models such as AdaBoost, KNN, Random Forest, Gradient Boosting, and XGBoost were benchmarked. For anomaly detection, techniques like Local Outlier Factor (LOF), Isolation Forest, and Autoencoders were employed to identify deviations indicative of non-compliance.

For the semi-supervised approach, we trained a deep neural network (DNN) following the principles of (Mandapati et al., 2023). To strictly avoid test leakage, the data was partitioned into disjoint sets: (i) a final test set used exclusively for evaluation, and (ii) a training set further divided into a labeled and an unlabeled portion. Approximately 80% of the training records retained their ground-truth labels, while the remaining 20% of the training set was treated as unlabeled. The semi-supervised DNN was optimized using a combined objective: a supervised cross-entropy loss applied to the labeled subset and an unsupervised consistency regularization loss applied to the unlabeled subset. This setup allowed the model to exploit structural patterns in the feature space without accessing the test set during training. The architecture consisted of three fully connected hidden layers (with 256, 128, and 64 units, respectively), ReLU activations, batch normalization, and dropout (rate = 0.3). Training was performed using the Adam optimizer (learning rate = 1*e*^−3^, batch size = 128) for up to 100 epochs with early stopping based on validation loss.

DNN emerged as the most effective classifier for predicting the validity of company records, outperforming other methods in both predictive performance and computational efficiency. The DNN consists of multiple layers of fully connected neurons (Riera et al., 2022). The architecture can be described as follows:


(1) $$\begin{array}{l} h_{1}=\sigma \left(W_{1}X+b_{1}\right), \end{array}$$

(2) $$\begin{array}{l} h_{2}=\sigma \left(W_{2}h_{1}+b_{2}\right), \end{array}$$

(3) $$\begin{array}{l} \vdots \end{array}$$

(4) $$\begin{array}{l} h_{L}=\sigma \left(W_{L}h_{L-1}+b_{L}\right), \end{array}$$

where *h*_*L*_ is the output of the last hidden layer, *X* is the input data, *W*_*i*_ are the weight matrices, *b*_*i*_ are the bias terms, and σ is the activation function (e.g., ReLU or sigmoid).

The output layer produces the predicted probabilities for classification (Mahbobi et al., 2023):


(5) $$\begin{array}{l} ŷ=\sigma \left(W_{\text{out}}h_{L}+b_{\text{out}}\right), \end{array}$$

where ŷ is the predicted label (for classification tasks, typically a vector of probabilities), and *W*_out_ and *b*_out_ are the weights and bias for the output layer.

The loss function used to train the DNN in a semi-supervised setting is a combination of labeled and unlabeled data (Njima et al., 2022). The loss function is typically a cross-entropy loss for the labeled data and a reconstruction loss (e.g., autoencoder-based) for the unlabeled data:


(6) $$\begin{array}{l} L=L_{\text{supervised}}+\text{λ}L_{\text{unsupervised}}, \end{array}$$

where *L*_supervised_ is the cross-entropy loss on the labeled data:


(7) $$\begin{array}{l} L_{\text{supervised}}=-\underset{i}{\sum }y_{i}\log\left({ŷ}_{i}\right), \end{array}$$

and *L*_unsupervised_ is the unsupervised reconstruction loss, which could be based on techniques like autoencoders or other anomaly detection methods for the unlabeled data.

The parameter λ controls the trade-off between the supervised and unsupervised components of the loss function.

The model is trained by minimizing the total loss function using gradient-based optimization algorithms such as stochastic gradient descent (SGD) or Adam:


(8) $$\begin{array}{l} \theta =\theta -\eta \nabla_{\theta }L, \end{array}$$

where θ represents the parameters of the network, η is the learning rate, and ∇_θ_*L* is the gradient of the loss function with respect to the parameters.

To further enhance the DNN's predictive capability, anomaly scores were introduced as an additional feature. These anomaly scores were generated using Feature-injected Anomaly Detection (FIAD) (Chen et al., 2025), an unsupervised anomaly detection method based on graph neural networks (GNNs) (Zhou et al., 2022) designed to identify deviations in both node attributes and structure.

FIAD operates through three key modules: feature injection (Xiao et al., 2021), reconstruction (Zhou et al., 2024b), and anomaly detection (Guan et al., 2024). The feature injection module directly incorporates anomalous information into the feature dimensions, enabling a finer-grained detection of anomalies. This approach allows the model to process anomalies across all nodes rather than focusing on specific subsets, improving the granularity of anomaly detection and enhancing the representation of subtle deviations. The reconstruction module leverages a shared encoder, an attribute decoder, and a structure decoder to encode the input data and reconstruct the original attribute and structural matrices. Anomaly detection is then performed by comparing the original and reconstructed matrices. Significant deviations between these matrices result in the generation of anomaly scores, where nodes exhibiting notable discrepancies are identified as anomalous.

Mathematically, the anomaly score (Liang et al. 2024) *S*_*i*_ for node *v*_*i*_ is computed as:


(9) $$\begin{array}{l} S_{i}=\alpha ||\left(A-Â\right)⊙{\text{Φ}}_{i}{||}_{2}^{2}+\left(1-\alpha \right)||\left(X-\hat{X}\right)⊙{\text{Θ}}_{i}{||}_{2}^{2}, \end{array}$$

where *A* and *X* represent the original adjacency and attribute matrices, Â and $\hat{X}$ are the reconstructed matrices, Φ_*i*_ and Θ_*i*_ are penalty terms for mismatches in structure and attributes, and α controls the balance between structural and attribute-based anomaly contributions.

Finally, SHAP analysis (Cakiroglu et al., 2024) was employed to interpret the model's predictions and identify the most influential features for each target variable. For invalid addresses, features like company age, employee count, and property ownership were key drivers.

### 3.3 Multi-objective optimization of XGBoost

To enhance the classification accuracy of company data, focal loss (Ross and Dollár, 2017) was integrated into the XGBoost model to address the class imbalance issue in binary classification tasks. Additionally, NSGA-II (Deb et al., 2002) (Non-dominated Sorting Genetic Algorithm II) was employed to optimize two conflicting objectives: model accuracy and interpretability. Specifically, the goal is to find an optimal trade-off between maximizing the area under the precision-recall curve (AUC-PR) and minimizing the number of trees in the model, thereby balancing predictive performance and model complexity (Sagi and Rokach, 2021).

Focal Loss is an extension of the traditional binary cross-entropy loss designed to address the class imbalance issue by giving more importance to hard-to-classify examples, particularly those from the minority class. The Focal Loss for binary classification is defined as:


(10) $$\begin{array}{l} FL\left(p_{t}\right)=-\alpha_{t}{\left(1-p_{t}\right)}^{\gamma }\log\left(p_{t}\right) \end{array}$$

where:

- *p*_*t*_ is the predicted probability for the true class. For the positive class, *p*_*t*_ is the predicted probability of the positive class.
- α_*t*_ is a balancing factor that gives more weight to the minority class.
- γ is the focusing parameter, which adjusts the rate at which easy examples are down-weighted.

The standard binary cross-entropy loss (Ho and Wookey, 2019) (Log Loss) is given by:


(11) $$\begin{array}{l} CE\left(y,ŷ\right)=-y\log\left(ŷ\right)-\left(1-y\right)\log\left(1-ŷ\right) \end{array}$$

where:

- *y* is the true label.
- ŷ is the predicted probability for the positive class.

Focal loss modifies the binary cross-entropy loss by multiplying it with the factor ${\left(1-p_{t}\right)}^{\gamma }$, which reduces the loss contribution from well-classified examples and focuses more on harder examples (Mahmoodi et al., 2024).

In this study, the NSGA-II algorithm was employed to optimize model hyperparameters under multiple objectives, balancing predictive performance and model complexity. The search space included parameters such as learning rate, maximum tree depth, and the number of estimators for ensemble-based models. A population size of 50 individuals and a maximum of 100 generations were used, with crossover and mutation probabilities set to 0.9 and 0.1, respectively. The algorithm was terminated either upon reaching the maximum number of generations or if the Pareto front showed no improvement over 20 consecutive iterations.

A solution *x*_1_ is said to *dominate* another solution *x*_2_ if:


(12) $$\begin{array}{l} f_{1}\left(x_{1}\right)\geq f_{1}\left(x_{2}\right)\;\text{and}\;f_{2}\left(x_{1}\right)\leq f_{2}\left(x_{2}\right) \end{array}$$

where *f*_1_ and *f*_2_ are two conflicting objectives, such as AUC-PR (accuracy) and the number of trees (interpretability).

The steps in the NSGA-II algorithm are as follows:

1. A random initial population *P*_0_ of *N* candidate solutions is generated. Each solution *x* ∈ *P*_0_ corresponds to a set of hyperparameters for the XGBoost model, e.g.,

x=(n_trees,max_depth,learning_rate).

2. Each candidate solution is evaluated on multiple objectives. The primary objectives are:

- Accuracy: Measured by the AUC-PR (Area Under the Precision-Recall Curve), denoted by *f*_1_(*x*), which is aim to maximize.

(13) $$\begin{array}{l} \text{Objective 1:}\;f_{1}=\text{AUC-PR}\left(XGBoost\right) \end{array}$$

- Interpretability: Measured by the number of trees in the model, denoted by *f*_2_(*x*), which is aim to minimize.

(14) $$\begin{array}{l} \text{Objective 2:}\;f_{2}=\text{Number of Trees}\left(XGBoost\right) \end{array}$$

For any two solutions *x* and *y*, we say that *x* *dominates* *y* (written *x*≺*y*) if:


$$\begin{array}{l} f_{1}\left(x\right)\geq f_{1}\left(y\right)\;\text{and}\;f_{2}\left(x\right)\leq f_{2}\left(y\right), \end{array}$$

with at least one of the inequalities being strict.

3. The population is partitioned into non-dominated fronts *F*_1_, *F*_2_, … as follows:

- *F*_1_ is the set of solutions that are not dominated by any other solution in the population.
- *F*_2_ consists of solutions that are dominated only by those in *F*_1_, and so on.

Each solution is assigned a Pareto rank *r*(*x*) based on the front it belongs to:


$$\begin{array}{l} r\left(x\right)=i\;\text{if}\;x\in F_{i}. \end{array}$$

4. To maintain diversity within each front, a *crowding distance* *d*(*x*) is computed for each solution *x* in a front *F*. For each objective *j* (with *m* objectives), let

$$\begin{array}{l} f_{j}^{\min}=\underset{x\in F}{\min}f_{j}\left(x\right),\;f_{j}^{\max}=\underset{x\in F}{\max}f_{j}\left(x\right). \end{array}$$

After sorting the solutions in *F* in ascending order according to *f*_*j*_(*x*), the crowding distance for an interior solution *x*_*i*_ is defined as:


$$\begin{array}{l} d\left(x_{i}\right)=\overset{m}{\underset{j=1}{\sum }}\frac{f_{j}\left(x_{i+1}\right)-f_{j}\left(x_{i-1}\right)}{f_{j}^{\max}-f_{j}^{\min}}, \end{array}$$

where *x*_*i*+1_ and *x*_*i*−1_ are the immediate neighbors of *x*_*i*_ in the sorted list for objective *j*. Boundary solutions are assigned an infinite (or very large) crowding distance to ensure their preservation.

5. Using a binary tournament selection process, solutions are chosen based on their Pareto rank (Tušar and Filipič, 2014) *r*(*x*) and crowding distance *d*(*x*). Specifically, a solution with a lower rank (i.e., belonging to a better Pareto front) and a larger crowding distance (i.e., located in a less crowded region) is preferred. Crossover and mutation operators are then applied to the selected individuals to generate an offspring population *Q*_*t*_.
6. After a predefined number of generations *T*, the final population *P*_*T*_ is obtained. The set of Pareto-optimal solutions, i.e., those on the first front *F*_1_ of *P*_*T*_, represents the trade-offs between maximizing AUC-PR and minimizing the number of trees:

$$\begin{array}{l} \text{Pareto-optimal set}=\left\{x\in P_{T}|x\text{is not dominated by any}y\in P_{T}\right\}. \end{array}$$

Focal Loss and NSGA-II were combined to optimize the hyperparameters of an XGBoost model, ensuring that both accuracy and interpretability are optimized simultaneously. Focal Loss was defined as a custom objective function for binary classification. The objective function for focal loss is:


(15) $$\begin{array}{l} L\left(y,ŷ\right)=-\alpha y{\left(1-ŷ\right)}^{\gamma }\log\left(ŷ\right)-\left(1-y\right){ŷ}^{\gamma }\log\left(1-ŷ\right) \end{array}$$

Here, *y* is the true label, and ŷ is the predicted probability of the positive class.

By combining Focal Loss and NSGA-II, this approach addresses the class imbalance problem using a custom loss function while simultaneously performing multi-objective optimization to balance the trade-off between accuracy (AUC-PR) and interpretability (number of trees). The result is a set of Pareto-optimal solutions that provide the best balance between these two conflicting objectives.

## 4 Results

**Figure 7:**
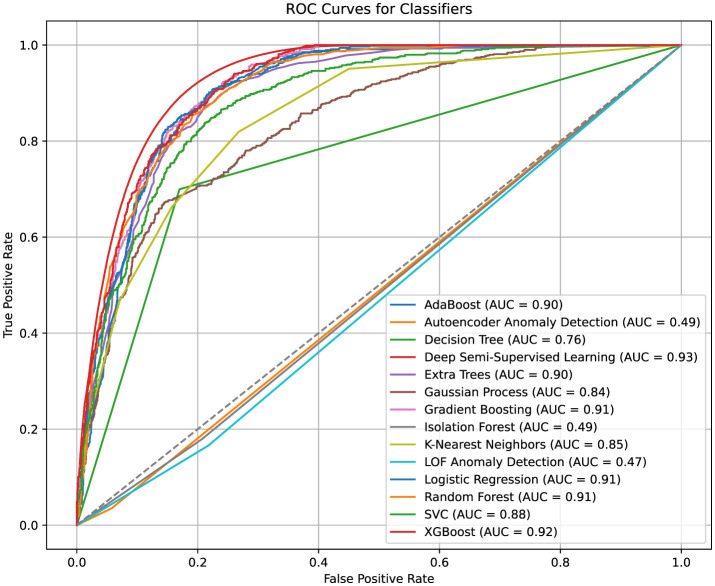
ROC AUC curves for models.

Figure 7 presents the ROC curves, illustrating the performance of ML classifiers without hyperparameter tuning. In all experiments, the data was split into 80% for training and 20% for testing. XGBoost and Deep Semi-Supervised Learning (DSSL) models achieved the highest AUC values, with scores of 0.92 and 0.93, respectively, demonstrating their strong discriminatory power. The robust performance of XGBoost, along with its interpretability through SHAP, made it the preferred classifier for this task.

Gradient Boosting and Random Forest models also showed competitive AUC scores of 0.91, indicating their capability to handle complex, nonlinear relationships within the data. Logistic Regression and Support Vector Classifier (SVC) performed well, with AUC values exceeding 0.88. However, they lacked the flexibility of tree-based methods in capturing feature interactions, which limited their performance in comparison.

Anomaly detection methods, such as Local Outlier Factor (LOF) and Isolation Forest, performed poorly, with AUC values around 0.49. This suggests that invalid address detection may not align well with their unsupervised anomaly-detection paradigms, which are typically more suited to different types of data anomalies.

In the results Table 2, the DSSL model achieved the highest F1 Score of 0.7994 ± 0.0133 (mean ± standard deviation across 5-fold cross-validation), indicating a strong balance between Precision (0.7645 ± 0.0162) and Recall (0.8377 ± 0.0148). Cross-validation was performed using StratifiedKFold with 5 splits, shuffling enabled, and a fixed random seed to ensure reproducibility and maintain class balance in each fold. For each fold, the model was trained on 80% of the data and evaluated on the remaining 20%, and all performance metrics were averaged across folds.

**Table 2:** Cross-validated performance metrics (mean ± std) of benchmark models.

**Model**	**AUC**	**Precision**	**Recall**	***F*_1_**	**Acc**.
AdaBoost	0.9019 ± 0.0123	0.7471 ± 0.0156	0.7613 ± 0.0172	0.7541 ± 0.0160	0.8295 ± 0.0105
Autoencoder	0.4888 ± 0.0254	0.2406 ± 0.0131	0.0352 ± 0.0085	0.0614 ± 0.0102	0.6324 ± 0.0206
Decision Tree	0.7649 ± 0.0182	0.6744 ± 0.0147	0.6996 ± 0.0195	0.6867 ± 0.0174	0.7865 ± 0.0129
DSSL	0.9303 ± 0.0098	0.7645 ± 0.0162	0.8377 ± 0.0148	0.7994 ± 0.0133	0.8558 ± 0.0107
Extra Trees	0.8968 ± 0.0135	0.7402 ± 0.0159	0.7671 ± 0.0170	0.7534 ± 0.0156	0.8275 ± 0.0112
Gaussian Process	0.8374 ± 0.0210	0.6888 ± 0.0171	0.6798 ± 0.0168	0.6842 ± 0.0164	0.7845 ± 0.0136
Gradient Boosting	0.9134 ± 0.0109	0.7439 ± 0.0142	0.8035 ± 0.0151	0.7726 ± 0.0139	0.8375 ± 0.0110
Isolation Forest	0.4861 ± 0.0225	0.3100 ± 0.0128	0.1795 ± 0.0104	0.2273 ± 0.0113	0.5832 ± 0.0185
KNN	0.8482 ± 0.0186	0.6884 ± 0.0160	0.6623 ± 0.0177	0.6751 ± 0.0165	0.7810 ± 0.0124
LOF	0.4741 ± 0.0231	0.2800 ± 0.0142	0.1657 ± 0.0098	0.2082 ± 0.0106	0.5740 ± 0.0192
Logistic Regr.	0.9088 ± 0.0115	0.7493 ± 0.0137	0.7728 ± 0.0152	0.7609 ± 0.0140	0.8375 ± 0.0111
Random Forest	0.9072 ± 0.0127	0.7555 ± 0.0148	0.7205 ± 0.0163	0.7376 ± 0.0150	0.8285 ± 0.0108
SVC	0.8826 ± 0.0132	0.7293 ± 0.0150	0.7060 ± 0.0158	0.7175 ± 0.0145	0.8090 ± 0.0113
XGBoost	0.9161 ± 0.0104	0.7362 ± 0.0141	0.7962 ± 0.0155	0.7650 ± 0.0136	0.8320 ± 0.0109

XGBoost and Gradient Boosting also showed strong F1 Scores of 0.7650 and 0.7726, respectively, indicating that these tree-based models effectively balance Precision and Recall (Uddin and Lu, 2024), even though they did not outperform the DSSL model. On the other hand, Autoencoder Anomaly Detection and Isolation Forest, with F1 Scores of 0.0614 and 0.2273, demonstrated significantly poorer performance, particularly in terms of Recall. These models struggled to capture positive instances, highlighting their limitations for the given task of detecting valid and invalid company records. AdaBoost, Logistic Regression, and Random Forest achieved moderate F1 Scores (around 0.75), showing good performance, but not as strong as the more advanced methods.

**Figure 8:**
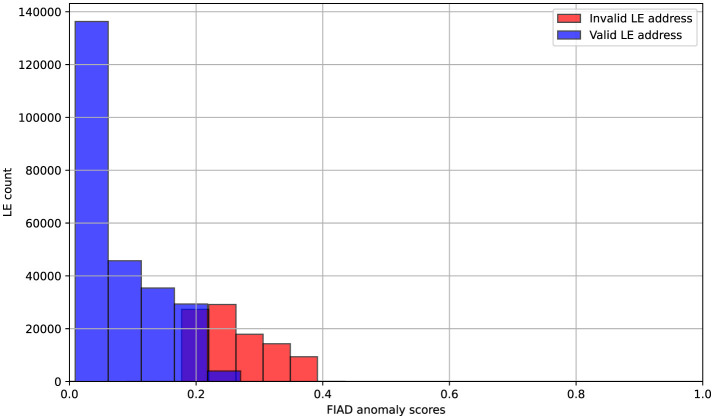
Distribution of FIAD anomaly scores in relation to the target class.

The inclusion of FIAD-generated anomaly scores in the DNN allowed the model to incorporate global patterns of deviation into its semi-supervised learning pipeline. For Figure 8 scores are normalized between 0 and 1. Higher scores indicate a greater likelihood of anomalies. Due to class imbalance in the target variable, the dataset was adjusted to ensure a balanced representation of both classes for the histogram, preventing skewed distributions. The histogram shows that normal data points are concentrated near 0, with their distribution predominantly in the range of 0 to 0.2. In contrast, anomalous data points are distributed in the range of approximately 0.18 to 0.38, demonstrating a clear separation between valid and invalid instances based on the anomaly score.

**Figure 9:**
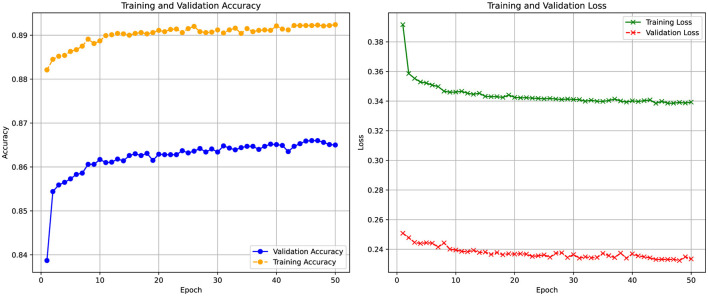
Training and validation accuracy (left) and loss (right) curves of the DSSL model over 50 epochs.

Figure 9 illustrates the training and validation accuracy, as well as the loss curves of the DSSL model over 50 epochs with incorporated FIAD scores as new featrue. The validation accuracy gradually increases from 0.82 to 0.86 and stabilizes, demonstrating effective learning. The training accuracy reaches 0.89, indicating good generalization without clear overfitting. Training loss decreases from 0.38 to 0.28, while validation loss stabilizes around 0.24, confirming consistent performance. However, the poorly separable data presents challenges in forming decision boundaries, and class imbalance may bias the model toward the dominant class, requiring further evaluation with metrics like precision, recall, and F1-score. Despite these challenges, the model achieves an accuracy of 86.58%, representing a 1.2% improvement over the version without the FIAD feature. In the context of a large dataset (e.g., one million entries, as in the present case), this improvement could potentially reduce the number of cases to be checked by an average of 12,000 to 13,000.

**Figure 10:**
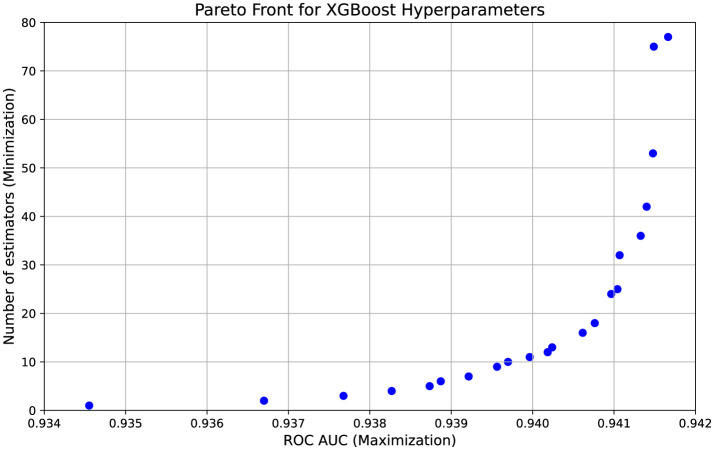
NSGA-II Pareto front.

The results of the NSGA-II optimization (Figure 10) demonstrate a clear trade-off between model accuracy, measured by ROC-AUC, and model complexity, represented by the number of trees in the XGBoost classifier. Given the complexity of the data, where company records are poorly separable and the dataset contains hundreds of thousands of entries, achieving a balance between interpretability and predictive performance is crucial. The highest ROC-AUC value obtained is 0.9417, consistently observed with 77 trees, indicating that this configuration provides the best predictive power. However, several alternative solutions with fewer trees achieve comparable performance, such as 0.9415 with 75 trees or 0.9413 with only 36 trees, suggesting that it is possible to maintain a high level of accuracy while improving model interpretability. The presence of models with significantly fewer trees, such as those with 25 or even 16 trees while still maintaining ROC-AUC values above 0.9400, highlights the potential for reducing model complexity without a severe drop in classification performance. At the same time, extreme cases, such as configurations with 1 to 4 trees that exhibit lower ROC-AUC values around 0.9367, confirm that reducing model complexity too aggressively results in a loss of predictive power. The optimization process successfully identifies a Pareto front of solutions, demonstrating that even a slight increase in classification accuracy can be meaningful given the scale of the dataset. In the context of tax authorities, where even marginal improvements can help reduce the number of unnecessary inspections.

**Figure 11:**
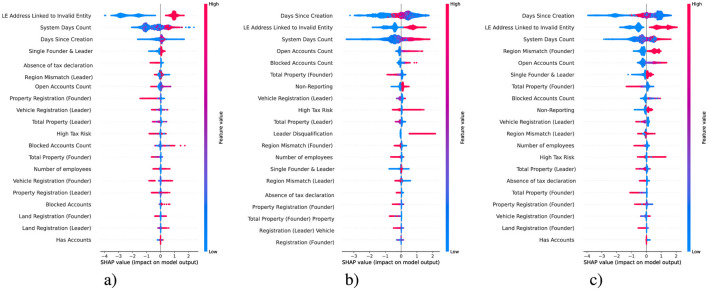
SHAP for **(a)** address, **(b)** leader, and **(c)** founder LE.

The SHAP summary plots in Figure 11 illustrate the influence of features on predicting the three target variables: the validity of address, leader information, and founder information. These plots identify the key factors contributing to discrepancies in the recorded data of the examined LEs. While the anomalous scores, introduced as part of the best-performing DSSL model, are not among the top-ranked features in terms of significance, their inclusion nevertheless improved the model's classification performance metric *F*_1_ by 1.1%. This improvement is noteworthy given the scale of the dataset, which consists of approximately one million records, highlighting the practical value of such an enhancement.

For address validity, the most impactful feature is the linkage of a company to an invalid entity. This suggests a strong association between past misuse of an address and its current reliability. Temporal factors, including the number of days the system has tracked the entity and the time since its creation, also influence. These variables likely capture behavioral patterns linked to compliance, with newer or older entities exhibiting distinct risks of irregularities (Hashmi et al., 2018). Other contributing factors, such as the absence of tax declarations and regional mismatches associated with leadership, may reflect discrepancies in operational practices or deliberate attempts to evade regulations.

In predicting leader information validity, temporal variables remain crucial, highlighting the persistent importance of an entity's lifecycle in identifying compliance risks. Factors such as leader disqualification, high tax risk, and non-reporting strongly impact predictions, which aligns with behaviors typically observed in problematic leadership practices. Regional mismatches and the concentration of ownership and leadership in a single individual further suggest vulnerabilities tied to operational scale and organizational structure, potentially enabling unchecked decision-making or reducing accountability (Liikanen et al., 2012).

For founder information validity, the analysis shows consistent relevance of temporal and operational factors, including the time since creation and the linkage of a company to an invalid entity. Specific founder-related features, such as mismatches in the founder's registered region and their property registrations, indicate potential red flags tied to founders' activities. These factors may point to intentional obfuscation of true operational details or failures to maintain accurate records. Additionally, issues linked to property and tax-related attributes reflect broader challenges in maintaining accurate documentation, especially in smaller or newer firms.

The interplay of organizational characteristics, temporal attributes, and individual roles impact in predicting non-compliance of companies key attributes. These findings are relevant for improving tax authority inspection systems to minimize unnecessary interventions.

## 5 Discussion

### 5.1 Key observations

The study presented several limitations and opportunities for future work. Firstly, the dataset used was not entirely representative of all LEs, as it focused only on younger companies with fewer than 100 employees. This selection may have limited the generalizability (Yaiprasert and Hidayanto, 2024) of the findings to larger or more established companies. Future studies could expand the dataset to include a broader range of entities, including those with a wider variety of ages and sizes, to better understand the applicability of the methods across different sectors.

Another limitation was the class imbalance in the dataset. While the imbalance was addressed through data filtration techniques, the challenge of separating valid and invalid records remained, especially for certain variables such as director and founder information (Balcaen and Ooghe, 2006). Future work could explore additional methods for handling imbalanced data, such as advanced oversampling or synthetic data generation techniques, to further improve model performance.

Additionally, the anomaly detection methods used, including the Local Outlier Factor (LOF) and Isolation Forest, performed poorly in this context. These methods, being unsupervised, struggled to identify invalid addresses effectively. This highlights the need for more robust anomaly detection techniques tailored to the characteristics of the dataset in scenarios involving complex relationships between the records. Future research could investigate hybrid approaches that combine unsupervised anomaly detection with supervised learning to improve performance (Nassif et al., 2021).

The application of multi-objective optimization (NSGA-II) enhances the practical applicability of the model in regulatory settings. Oerly complex models can be difficult to interpret and deploy in real-world compliance monitoring systems. By reducing the number of trees, the model remains computationally efficient and transparent, making it more suitable for practical use by tax authorities. However, some limitations should be considered. First, while Focal Loss addresses class imbalance effectively, alternative approaches such as oversampling, undersampling, or cost-sensitive learning (Fernández et al., 2018) could be explored. Second, NSGA-II, while effective, is computationally expensive (Verma et al., 2021), especially when optimizing over large search spaces. Future research could investigate alternative metaheuristic optimization techniques or hybrid approaches combining reinforcement learning with evolutionary strategies.

Finally, the inclusion of anomaly scores, while it improved model performance, was not among the most influential features in the SHAP analysis. This indicates that the potential of anomaly detection techniques might not be fully realized in this context. Future studies could explore more sophisticated methods for anomaly score generation and investigate how different anomaly detection techniques impact model interpretability and performance.

### 5.2 Future work

In future work, additional features could be incorporated to further improve the model's predictive power. For instance, financial data related to taxes, such as detailed tax payment history and discrepancies in reported taxes, could be useful in identifying patterns of non-compliance (Doran, 2009). Information regarding the size and type of the enterprise–whether it is a small or large company, and its industry sector–could provide valuable context for distinguishing between legitimate and potentially fraudulent entities.

The analysis was restricted to newly registered firms ( ≤ 3 years old), which are generally associated with higher compliance risks and thus represent a relevant target group for tax monitoring. While this focus allowed the study to capture early-stage anomalies in company records, it also limits the direct applicability of the results to older and larger firms. Validation on broader company populations, as well as across different time periods or jurisdictions, constitutes an important direction for future research to strengthen the generalizability of the proposed approach.

The evaluation metrics in this study were reported using standard measures such as ROC-AUC, Precision, Recall, and F1 score, without explicit optimization of thresholds in relation to audit costs. However, in practice, the relative importance of false positives and false negatives is determined by policy priorities (Kang and Wu, 2023), as unnecessary audits impose resource burdens while undetected fraudulent firms result in revenue losses. Future work should therefore consider cost-sensitive evaluation and threshold tuning, for example by analyzing the Precision-Recall curve (Boyd et al., 2013) or employing weighted utility functions (Ko et al., 2014). Such an extension would more directly align the machine learning outputs with real-world audit decision-making processes.

A promising direction for further research is the integration of anomaly detection methods or ensemble strategies with the DNN (Lei et al., 2022). Combining the DSSL model with XGBoost, for instance, could leverage the high recall of the neural network together with the interpretability and stability of tree-based models, thereby improving both accuracy and transparency. In addition, the application of model-agnostic interpretability tools such as SHAP directly to the DSSL outputs (Parisineni and Pal, 2024), or the use of surrogate models, may illustrate decision mechanisms while preserving predictive performance.

Furthermore, additional details about affiliated parties, particularly the family members of the founders, could enhance the model. This could include information about the relationships between founders and their relatives, as well as the assets they own, such as real estate. Expand the feature set to include financial indicators [e.g., tax payment history (Brühne and Schanz, 2022), VAT claims (Van Brederode, 2008), revenue consistency (Qian and Li, 2013; Wright, 2016)], firm attributes (Salehi et al., 2022; Mathew et al., 2016; Alabdullah et al., 2021) (industry sector, size, age) and relational data (ownership links, affiliated parties). Financial and sectoral variables provide direct signals of misreporting and improve the model's capacity to distinguish legitimate variance from anomalous behaviour. Relational features and graph-derived metrics enable detection of complex schemes such as shell-company networks or proxy ownership (Paligorova, 2010) that are not visible from single-entity records. Integrating external sources (credit ratings, public records, media reports) would further contextualize internal tax data and is expected to increase predictive accuracy and reduce false positives in audit targeting.

Moreover, indicators related to taxation, such as VAT deductions, could be integrated into the model (Jenkins and Kuo, 2000). For example, a company with unusually low VAT deductions, compared to industry standards or expectations set by the tax authority, may signal potential non-compliance or fraudulent activities. These new data points would provide a more comprehensive view of an entity's financial behavior and could further strengthen the model's ability to detect inaccurate or fraudulent records.

## 6 Conclusions

This study explored the application of ML techniques to identify inaccuracies in company data using records from the Federal Tax Service of Russia. Various approaches, including supervised learning, anomaly detection, and semi-supervised learning, were applied to effectively classify and validate key information such as addresses, director details, and founder data. Key findings can be summarized as it follows:

- By refining the dataset to exclude older companies and large organizations, the class balance was improved, which subsequently enhanced the model's accuracy.
- The inclusion of FIAD scores led to a 1.2% increase in classification accuracy, improving the model's ability to detect deviations.
- Key predictive features included company age and temporal factors, which played a key role in identifying compliance risks.
- SHAP analysis revealed crucial features, such as regional mismatches and anomalies in ownership structure, as primary indicators of potential non-compliance.
- DSSL model achieved the highest results with an AUC of 0.9303 and an F1-score of 0.7994.
- XGBoost with NSGA-II optimized hyperparameters achieved an AUC of 0.9417 using 77 trees.
- The improved classification accuracy will assist tax authorities in reducing unnecessary inspections and enhancing the efficiency of tax compliance processes.

## Data Availability

The original contributions presented in the study are included in the article/supplementary material, further inquiries can be directed to the corresponding author.
